# Multidisciplinary Management of Pediatric Craniopharyngioma: From Diagnosis to Long-Term Outcomes

**DOI:** 10.3390/biomedicines14081808

**Published:** 2026-08-11

**Authors:** Lucia Quaglietta, Francesca Vitulli, Carmela Russo, Anna Grandone, Sabina Vennarini, Maria Elena Errico, Francesco Tengattini, Pietro Spennato, Nicola Onorini, Stefania Picariello, Lucia de Martino, Nicola Improda, Antonella Klain, Pier Paolo Panciani, Eugenio Maria Covelli, Giuseppe Cinalli

**Affiliations:** 1 Neurooncology Unit, Department of Oncology, Hematology and Cellular Therapy, Santobono-Pausilipon Children’s Hospital, AORN, IRCCS, 80129 Naples, Italy; 2 Neurosurgery Unit, Department of Neurosciences, Santobono-Pausilipon Children’s Hospital, AORN, IRCCS, 80129 Naples, Italy; 3 Neuroradiology Unit, Department of Neurosciences, Santobono-Pausilipon Children’s Hospital, AORN, IRCCS, 80129 Naples, Italy; c.russo1@santobonopausilipon.it; 4 Department of Woman, Child and General and Specialized Surgery, University of Campania “Luigi Vanvitelli”, 80138 Naples, Italy; 5 Pediatric Radiotherapy Unit, Fondazione IRCCS Istituto Nazionale Tumori, 20133 Milan, Italy; 6 Pathology Unit, Santobono-Pausilipon Children’s Hospital, AORN, IRCCS, 80129 Naples, Italy; 7 Neurosurgery Unit, Department of Medical and Surgical Specialties, Radiological Sciences and Public Health, University of Brescia, 25121 Brescia, Italypierpaolo.panciani@unibs.it (P.P.P.); 8 Neuro-Endocrine Diseases and Obesity Unit, Department of Neurosciences, Santobono-Pausilipon Children’s Hospital, AORN, IRCCS, 80129 Naples, Italy; n.improda@santobonopausilipon.it; 9 Auxo-Endocrinology Unit, Santobono-Pausilipon Children’s Hospital, AORN, IRCCS, 80129 Naples, Italy

**Keywords:** adamantinomatous craniopharyngioma, papillary craniopharyngioma, CTNNB1 mutation, BRAF V600E, hypothalamus-sparing strategies, pediatric skull base surgery, proton beam radiotherapy, long-term outcomes, quality of life

## Abstract

**Background**: Pediatric craniopharyngiomas are rare, histologically differentiated epithelial tumors arising in the sellar–suprasellar region. Despite their low-grade classification, these tumors are associated with significant long-term morbidity because of their proximity to critical neurovascular and hypothalamic structures. Management remains challenging and requires a careful balance between durable tumor control and the preservation of neurological, endocrine, and neurocognitive functions. **Methods**: This narrative review summarizes the current evidence concerning the epidemiology, molecular pathogenesis, clinical presentation, diagnostic evaluation, and treatment strategies for pediatric craniopharyngiomas. Emphasis is placed on anatomical and hypothalamic involvement-based classifications, evolving surgical philosophies, and the role of multimodal management. **Results**: Advances in molecular characterization have resulted in the delineation of two distinct subtypes—adamantinomatous and papillary craniopharyngiomas—with different biological drivers and therapeutic implications. Surgical management has evolved from historically aggressive gross total resection to risk-adapted, anatomy-driven approaches that prioritize hypothalamic preservation. In selected patients, subtotal resection combined with modern radiotherapy provides tumor control comparable to that of radical surgery, with reduced morbidity. Endoscopic endonasal and microsurgical transcranial approaches play complementary roles, with approach selection guided by tumor anatomy, hypothalamic involvement, and surgical expertise. Emerging targeted therapies and intracystic treatments further expand therapeutic options. **Conclusions**: The contemporary management of pediatric craniopharyngiomas emphasizes individualized, multidisciplinary strategies focused on functional preservation rather than anatomical radicality alone. Integration of molecular insights, advanced imaging, and long-term outcome data is essential to further improve survival and quality of life in this vulnerable population.

## 1. Introduction

Craniopharyngiomas are rare, benign epithelial tumors with solid or mixed solid-cystic components that originate from embryonic remnants of Rathke’s pouch [1] and are referred to as “Rathke’s pouch tumors” or “pituitary duct tumors”; these lesions are typically located in the sellar or suprasellar region and arise along the pituitary–hypothalamic axis [2]. Their proximity to critical structures such as the pituitary gland, hypothalamus, and optic chiasm presents unique challenges in children [3]. Despite their WHO Grade I classification and benign histology [4], craniopharyngiomas can exhibit clinically aggressive behavior because of local invasion and treatment-related sequelae. These tumors have a substantial effect on long-term quality of life. They frequently recur over the course of a patient’s lifetime and require a multidisciplinary approach because of their polymorphic clinical presentation [5].

## 2. Epidemiology

The annual incidence of craniopharyngioma is estimated to be 0.5–2.0 new cases per million people [6]. While they can occur at any age, craniopharyngiomas are traditionally considered pediatric tumors, accounting for 1.2–4% of all childhood intracranial neoplasms, with approximately 30–50% of cases diagnosed between 0 and 14 years of age [7]. In the United States, approximately 338 new cases are diagnosed annually, including 96 in children aged 0–14 years [7]; a comparable estimate is 350 cases per year, with an age-adjusted incidence of 0.19 per 100,000 individuals [8]. The age distribution is bimodal, with a first peak between ages 5 and 14 [9] and a second between 50 and 74 years [6,10]. No significant differences in incidence have been reported by sex, race, or geographic region, although the Central Brain Tumor Registry of the United States (CBTRUS) registry data suggest a slightly higher incidence among African American patients without sex-based differences. Familial cases are exceedingly rare.

The recurrence rate is approximately 50%, necessitating long-term surveillance. Despite the high frequency of sequelae, overall survival is favorable, with a 91% 5-year survival rate and an 87% 10-year survival rate [3]. Adamantinomatous craniopharyngiomas (ACPs) predominate in children, whereas the papillary subtype (PCP) is more common in adults [4].

## 3. Pathogenesis, Classification, and Subtypes

Craniopharyngiomas typically arise along the pituitary stalk in the suprasellar region adjacent to the optic chiasm. A minority may be located within the sella turcica [11] and, in rarer cases, along the optic tract or within the third ventricle [12,13]. Although histologically differentiated, craniopharyngiomas are locally invasive and are thus considered low-grade malignant tumors. Most of these exhibit both solid and cystic components; cysts often contain a turbid fluid rich in cholesterol crystals.

Beyond histological classification, anatomical patterns of tumor growth and, in particular, the degree of hypothalamic involvement have emerged as key determinants of clinical behavior and treatment strategy. Several classification systems have been proposed to describe the relationship between the tumor and the hypothalamus. De Vile et al. [14] first introduced an MRI-based classification according to the degree of hypothalamic involvement, highlighting its prognostic relevance for postoperative morbidity. This concept was subsequently refined by Müller et al. [15], who developed a standardized staging system based on tumor extension along the sellar–suprasellar–third ventricular axis and its relationship with the hypothalamus. Other widely used systems include the topographical classification by Kassam [16], which is based on the tumor’s position relative to the optic chiasm and infundibulum, and the Puget grading system [17], which stratifies tumors according to the extent of hypothalamic involvement. These classifications have proven clinically relevant, as hypothalamic involvement strongly correlates with surgical risk, postoperative morbidity, and long-term functional outcomes [16,17].

In accordance with the 2021 WHO classification of central nervous system tumors, craniopharyngiomas are classified as ACP or PCP [4], and these tumors, which were previously considered histological subtypes of the same entity, clearly have distinct histopathological features, molecular alterations, methylation profiles, and age distributions.

**Figure 1:**
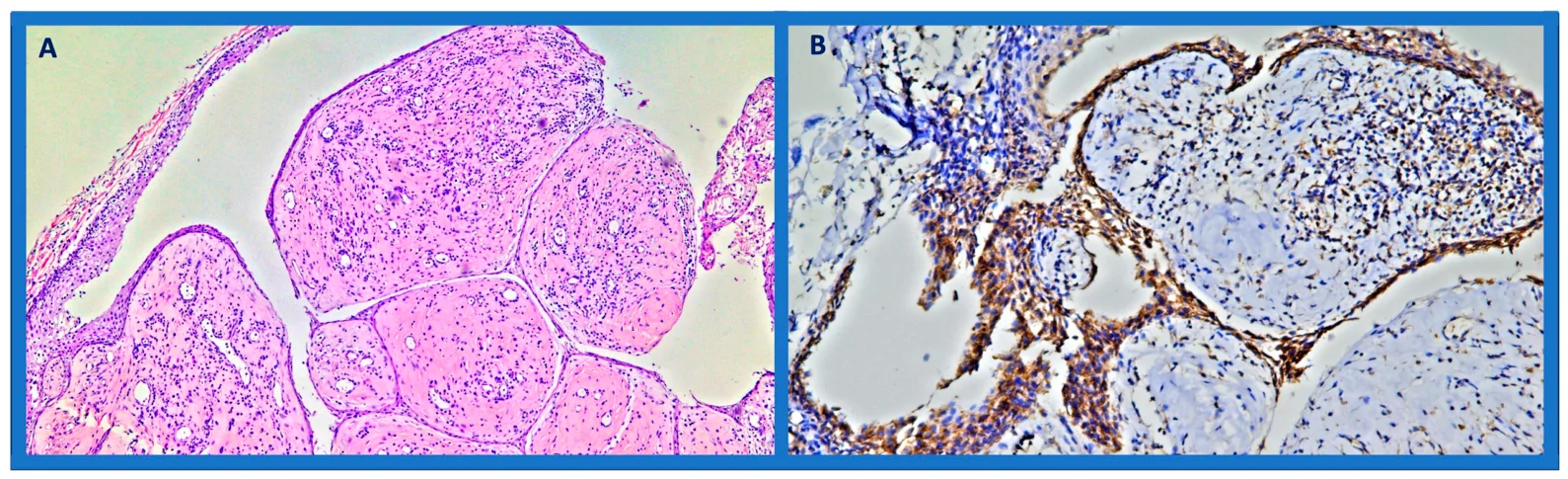
(**A**) Fibrovascular cores with nonkeratinizing squamous epithelium. H&E X100. (**B**) Cytoplasmic immunohistochemical staining for BRAF p.V600E. X200.

ACP is the predominant subtype, accounting for approximately 90% of cases, and is most commonly found in children. It is characterized by cystic and solid epithelial components, calcifications, solid tissue, and keratin nodules [3]. PCP occurs almost exclusively in adults and accounts for approximately 10% of cases [5]. Histologically, the neoplasm consists of a squamous, nonkeratinizing epithelium lining a fibrovascular core (Figure 1).

**Figure 2:**
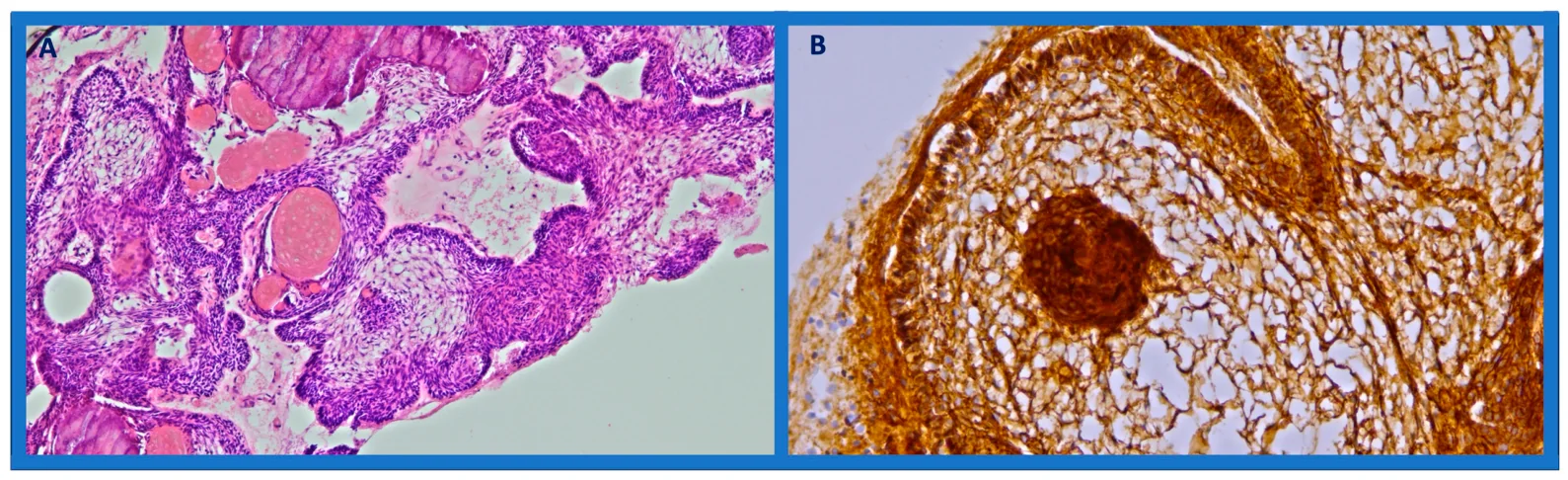
(**A**) Epithelial islands with basal palisading, wet keratin (anucleate ghost cells), and calcifications. H&E X100. (**B**) Plurifocal nuclear immunoreactivity for beta-catenin. X400.

Pathologically, ACP is defined by somatic activating mutations in the CTNNB1 gene encoding β-catenin [18]. These mutations, which are typically located on exon 3 (the degradation domain), prevent β-catenin degradation, resulting in its cytoplasmic and nuclear accumulation [19,20]. This leads to constitutive activation of the Wnt/β-catenin signaling pathway, a key oncogenic driver of ACP [20]. Histologically, this manifests as nuclear β-catenin positivity in cell clusters within the tumor (Figure 2), promoting the transcription of pro-proliferative target genes such as AXIN2, LEF1, and BMP4. In addition to Wnt pathway activation, Sonic Hedgehog (SHH) signaling is hyperactive, particularly in palisading tumor cells and proliferative cell clusters. ACPs are also characterized by significant intratumoral heterogeneity: only a subpopulation of SOX2-positive cells harbor CTNNB1 mutations and function as signaling centers, secreting growth factors such as VEGF, EGF, FGF, TGF-β, and SHH. These factors act via paracrine mechanisms on neighboring nonmutated cells in a process consistent with the senescence-associated secretory phenotype (SASP). ACPs also display a distinct epigenetic signature, including a unique DNA methylation pattern that differentiates them from PCPs.

Conversely, PCPs exhibit a homogenous molecular profile, with 90–95% harboring the BRAF V600E mutation (valine-to-glutamate substitution in BRAF) [18]. This mutation leads to constitutive activation of BRAF and the MAPK/ERK pathway, increasing cell proliferation and survival. Unlike ACPs, β-catenin in PCPs remains localized to the membrane without activation of the Wnt pathway.

**Figure 3:**
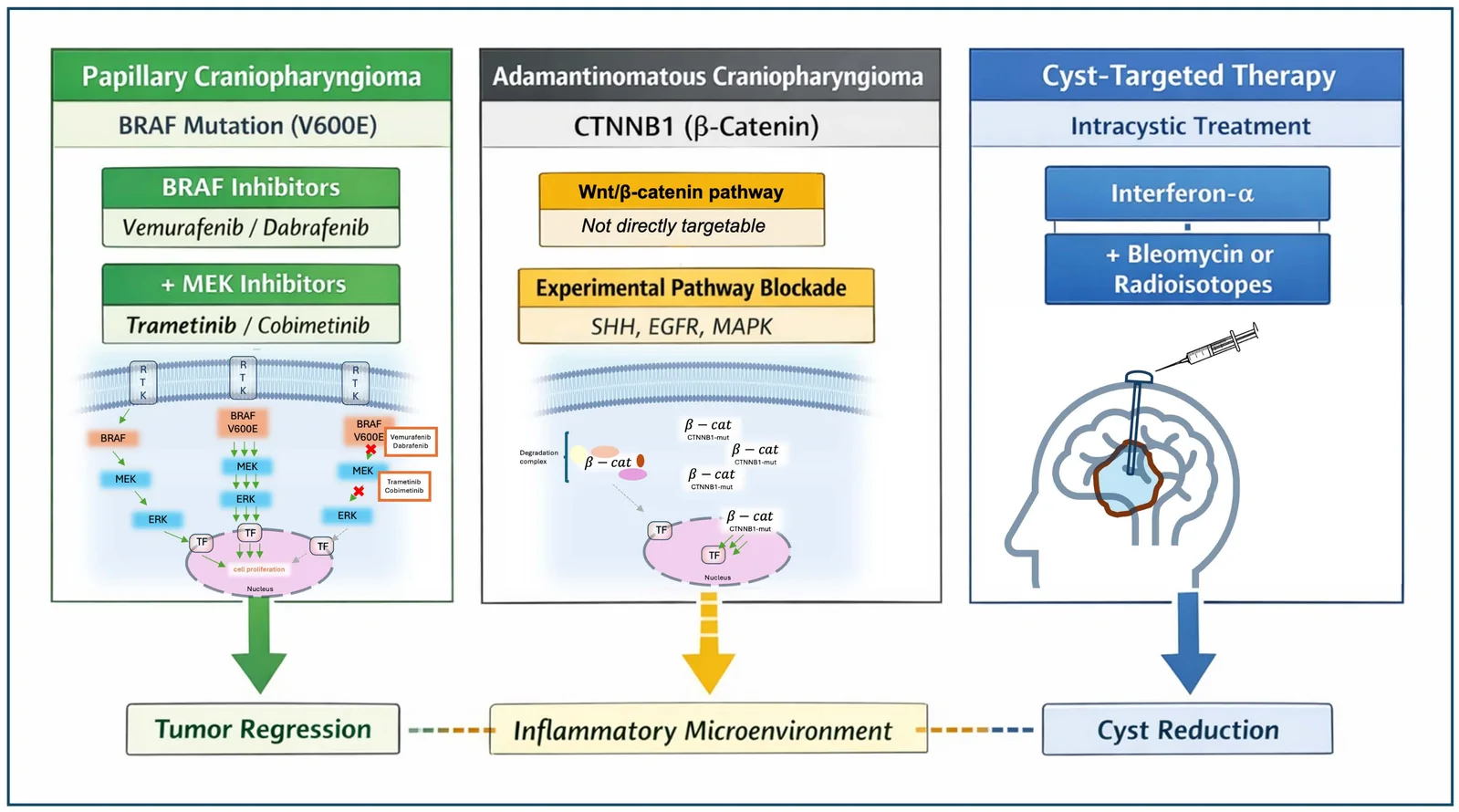
Molecularly and biologically driven therapies for craniopharyngiomas. Papillary craniopharyngiomas are characterized by the activation of BRAF V600E mutations, leading to constitutive activation of the MAPK/ERK signaling pathway. In this subtype, targeted inhibition with BRAF inhibitors alone or in combination with MEK inhibitors can effectively suppress downstream signaling and induce marked tumor regression. In contrast, adamantinomatous craniopharyngiomas harbor activating CTNNB1 mutations with aberrant Wnt/β-catenin signaling, which is not directly targetable. Current investigational strategies focus on indirect modulation of downstream or associated pathways and on the inflammatory tumor microenvironment. In both subtypes, particularly in tumors with a predominant cystic component, intracystic therapies—most commonly interferon-α, with bleomycin or radioisotopes reserved for selected cases—can achieve local cyst reduction and symptom relief while minimizing systemic toxicity.

Immunohistochemical staining for BRAF V600E (VE1 antibody) is characteristically positive (Figure 3). Genomically and epigenetically, PCPs are homogeneous, with BRAF mutations typically clonal and uniformly distributed. Transcriptional profiles indicate MAPK pathway activation and the expression of genes related to Rathke’s pouch tumor development. The PCP methylome also forms a distinct epigenetic cluster separate from that of ACPs [3,4,5,11,12,13,18,19,20].

## 4. Clinical Presentation

The clinical presentation of pediatric craniopharyngioma is often insidious and heterogeneous, resulting in a prolonged duration of symptoms before diagnosis. The median duration of history has been reported to be approximately 6 months, although it may be considerably longer in some patients [21].

The tumor is located in the sellar/suprasellar region, with potential extension to the optic chiasm, hypothalamus, third ventricle, and pituitary gland. Clinical variability is influenced by patient age, histological subtype, and hypothalamic involvement [17,22,23,24]. In fact, lesions involving the mammillary bodies and posterior hypothalamus frequently trigger hypothalamic syndrome (HS), negatively impacting on the patient’s long-term prognosis [3,5].

Initial symptoms are often nonspecific and include headache, nausea, vomiting, growth retardation, and disturbances of pubertal development (precocious or delayed puberty). Endocrine disturbances are among the earliest and most frequent findings and are attributed to disruption of the hypothalamic–pituitary axis: more than 70% of children present with growth deceleration and delayed puberty [25]; arginine vasopressin deficiency (AVP-D)—manifesting as polyuria and polydipsia—is a prevalent symptom, with an incidence of 14–18% before operation [26]. Central hypothyroidism and adrenal insufficiency are also common in the preoperative phase and can lead to fatigue, weight gain, hypoglycemia, hypotension, and electrolyte imbalance [27,28]. Between 12% and 19% of patients are already obese at the time of their craniopharyngioma diagnosis [27,28]. Less common endocrine abnormalities include hypogonadism (with amenorrhea, gynecomastia, or infertility) and hyperprolactinemia (resulting in galactorrhea).

Visual disturbances occur in 50–80% of cases at diagnosis [29], often due to optic chiasm compression. Bitemporal hemianopsia is the most typical finding, reported in 60–84% of patients [29,30], and is frequently associated with reduced visual acuity, diplopia [29,31], blurred vision, altered color perception, and papilledema in cases of increased intracranial pressure [28]. A comparative analysis of 310 patients highlighted age-related and tumor extension-dependent differences in visual impairment [31]. Nystagmus or strabismus may occur with cranial nerve involvement.

Symptoms related to mass effects and intracranial hypertension are common, particularly in tumors extending into the third ventricle. Headaches, often frontal and positional, are reported in approximately 50% of patients [30,32] and may be accompanied by vomiting and somnolence because of obstructed cerebrospinal fluid flow [28]. In severe cases, altered consciousness and, rarely, seizures can occur—especially with cortical extension or hydrocephalus [29]. Spontaneous rupture of cystic components may result in chemical meningitis or temporary symptom relief [33].

Neuropsychological and behavioral symptoms may include hypothalamic-related obesity, hyperphagia, hypersomnia, disordered sleep, apathy, irritability, impulsiveness and depression. Cognitive impairment, especially involving memory and attention, is common in lesions extending toward the third ventricle [3,34,35].

Neuroradiological Work-up and Preoperative Assessment Imaging plays a crucial role in the diagnosis and management of craniopharyngiomas, with MRI (magnetic resonance imaging) on high-field scanners (1.5 Tesla or higher) serving as the gold standard for evaluating these sellar-region lesions. Standard protocols include T1- and T2-weighted sequences in the sagittal and coronal planes with thin slices, and gadolinium contrast administration is recommended to characterize these neoplasms. Advanced sequences such as diffusion-weighted imaging (DWI), susceptibility-weighted imaging (SWI), volumetric imaging, and perfusion-weighted imaging (PWI) can provide additional information about lesion characteristics and help differentiate craniopharyngiomas from other sellar masses. In particular, three-dimensional high-resolution T2-weighted sequences and balanced steady-state free precession (bSSFP) sequences (also known as CISS, FIESTA or 3D DRIVE) play important roles, as they better delineate the pituitary stalk, suprasellar cistern, and cystic lesions, which are essential for surgical planning [36]. DWI provides information about lesion cellularity by measuring water molecule diffusion movements, and recent studies have shown that apparent diffusion coefficient (ADC) values correlate with tumor consistency and the proliferative index, even if these data require further validation. On the other hand, SWI is useful for depicting intralesional calcification or hemorrhage, allowing distinction between these entities on the basis of evaluation of both magnitude and phase maps [37]. PWI can provide additional information that is helpful for the differential diagnosis of sellar/parasellar tumors. Indeed, PWI allows the differentiation of craniopharyngiomas from other sellar tumors, such as pilocytic astrocytomas or meningiomas, which in some cases might be mimickers on conventional MR scans [38]. Finally, magnetic resonance angiography (MRA) can be performed with or without contrast administration and defines the size and course of intracranial arteries, which may be displaced and compressed by a sellar lesion.

While MRI provides superior soft-tissue detail, CT remains valuable for confirming calcifications, frequently showing an egg-shell pattern around the cystic component, which is present in 70–90% of cases and represents a key diagnostic feature [39], and for assessing bony anatomy, particularly the degree of aeration of the sphenoid sinus, when a transnasal surgical approach is considered.

The two subtypes of craniopharyngiomas exhibit different imaging characteristics.

**Figure 4:**
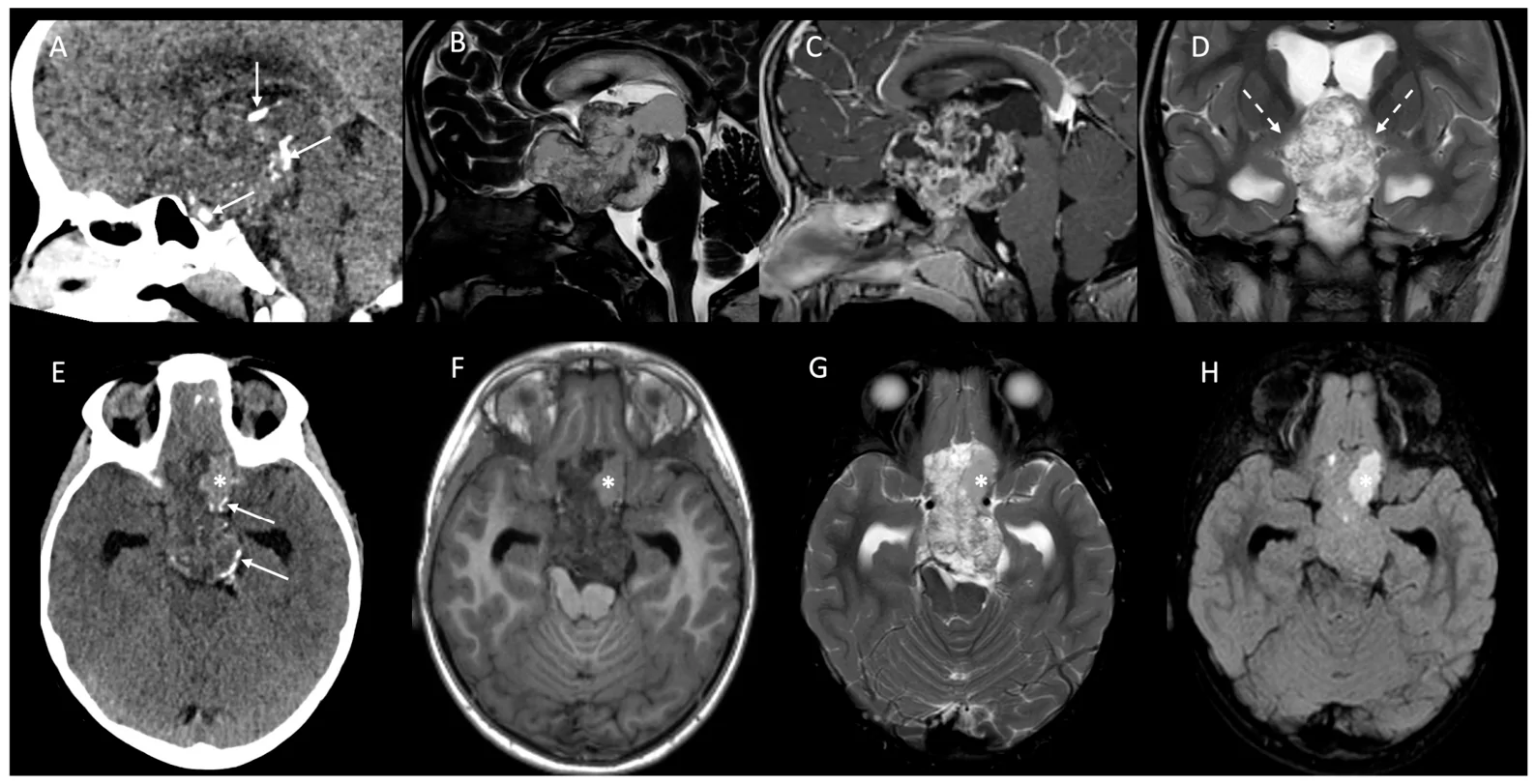
ACP. CT and MR studies of a 7-year-old boy with headache and vomiting. (**A**) Sagittal CT, (**B**) sagittal bSSFP, (**C**) sagittal contrast-enhanced 3D T1, (**D**) coronal T2, (**E**) axial CT, (**F**) axial 3D T1, (**G**) T2, and (**H**) FLAIR. Multiloculated sellar/suprasellar solid/cystic lesions involving the third ventricle with obstructive hydrocephalus and compression of the hypothalamus (**D**, dashed arrows). The lesion is characterized by inhomogeneous cystic content and strong enhancement of solid components and cystic walls. Note multiple intratumoral and peripheral calcifications (**A**,**E**, arrows) and the presence of hyperdense and T1/T2/FLAIR hyperintense proteinaceous cystic fluid (**E**–**H**, asterisks).

**Figure 5:**
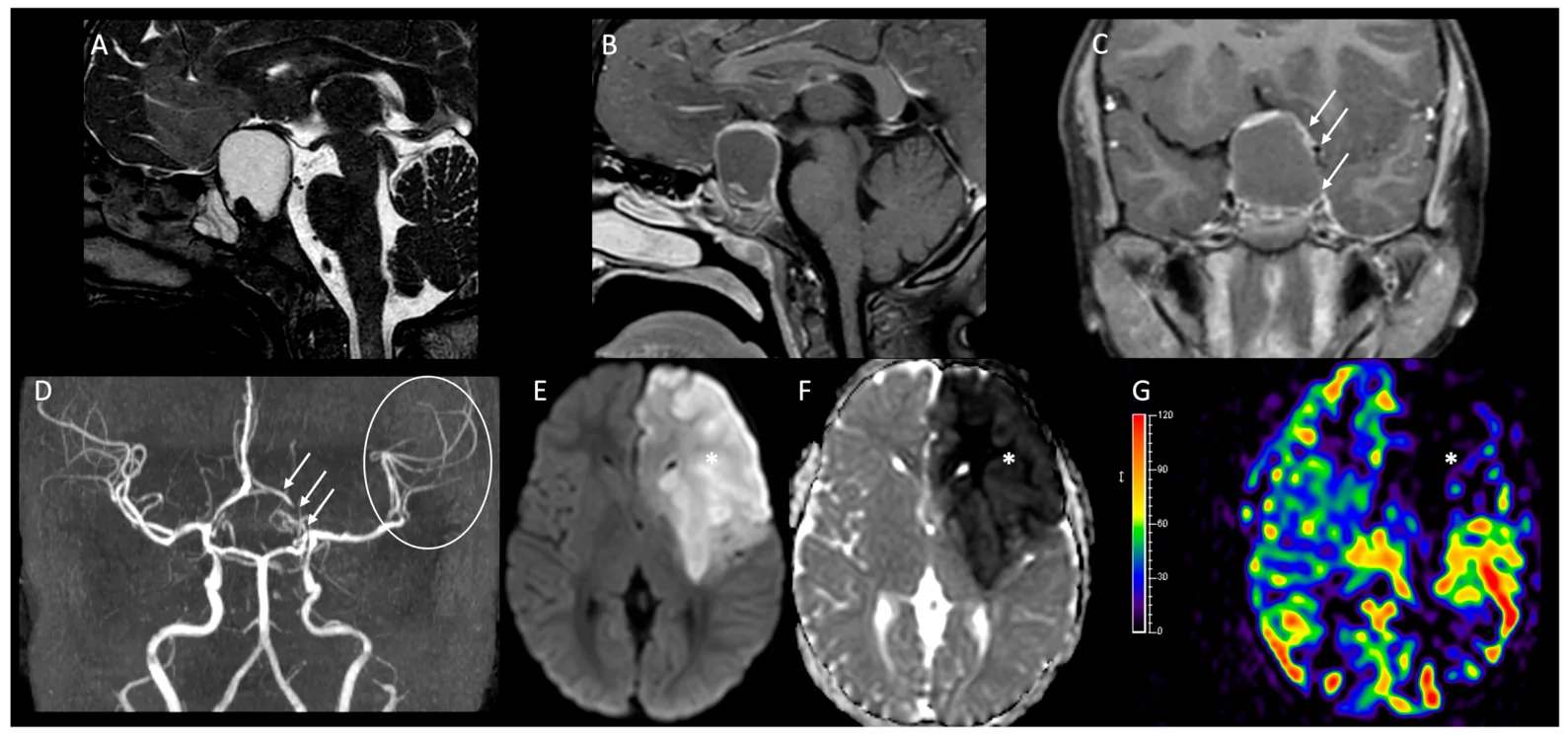
ACP. MRI/MRA study of a 6-year-old girl with right hemiparesis and vomiting. (**A**) Sagittal bSSFP, (**B**) sagittal and (**C**) coronal contrast-enhanced 3D T1, (**D**) coronal MIP (maximum intensity projection) reconstruction of 3D TOF (time-of-flight) MRA, (**E**) axial DWI, (**F**) axial ADC map, and (**G**) axial color-coded map of the PCASL (pseudocontinuous arterial spin labeling). Intra- and suprasellar lobulated solid/cystic lesions characterized by inhomogeneous content and peripheral enhancement (**A**–**C**). The lesion compresses the supraclinoid segment of the left internal carotid artery and its bifurcation with a reduced flow signal of the A1 segment of the left anterior cerebral artery (ACA) (**C**,**D**, arrows) and the M2–M3 segments of the left middle cerebral artery (MCA) (**D**, oval). Note the large area of cytotoxic edema on DWI/ADC (**E**,**F**) and reduced perfusion in the PCASL (**G**) involving the left fronto-insular and basal ganglia regions, which is consistent with ischemia of the left ACA and MCA territories (**E**–**G**, asterisks).

**Figure 6:**
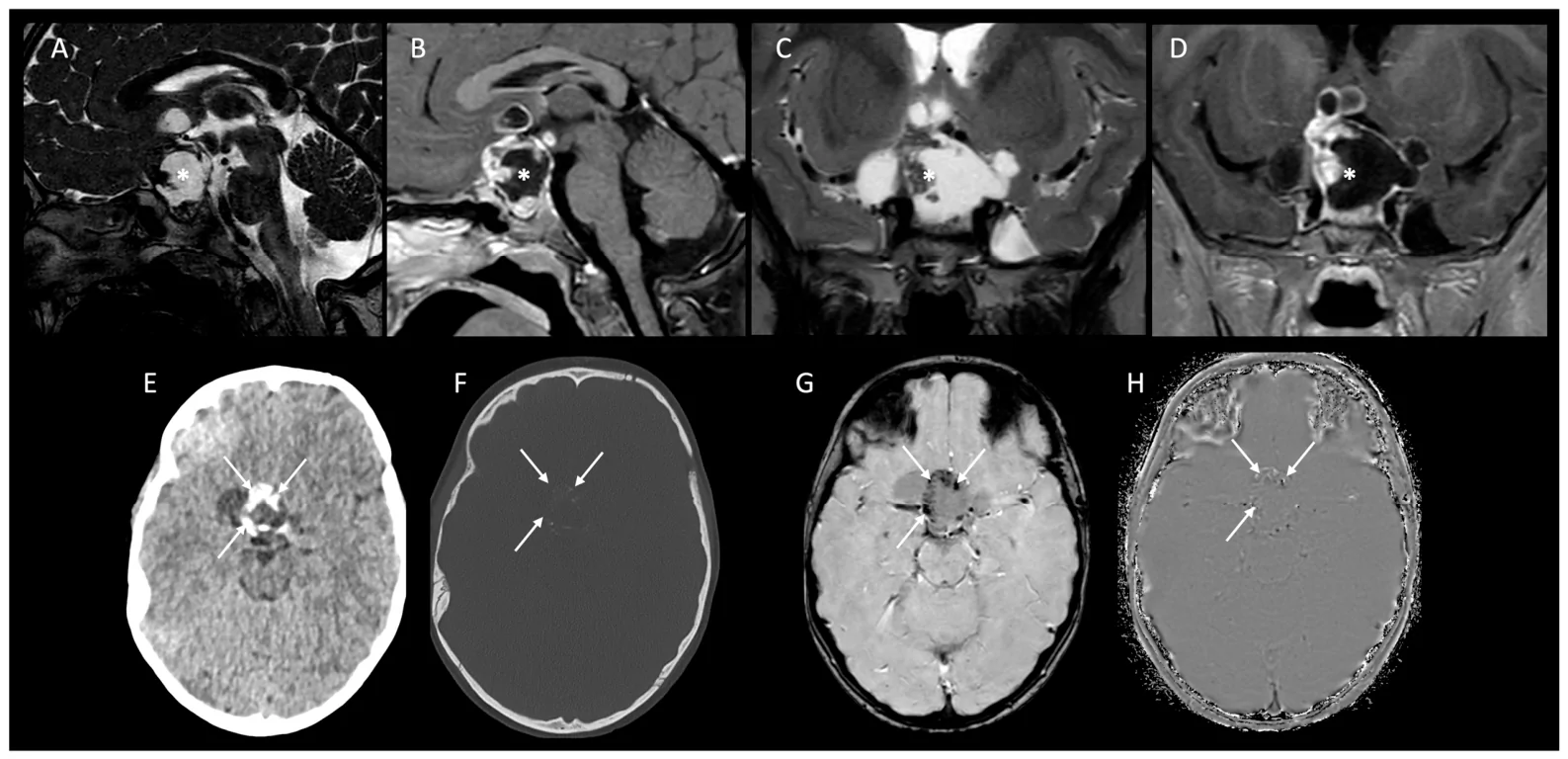
ACP. MRI and CT studies of a 5-year-old boy with headache and vomiting. (**A**) Sagittal bSSFP, (**B**) sagittal and (**C**) coronal contrast-enhanced T1, (**D**) coronal T2, (**E**) axial CT, (**F**) axial bone CT, (**G**) axial magnitude and (**H**) phase maps of SWI. Multiloculated cystic lesions involving the sellar and suprasellar cistern and bilateral anterior and middle cranial fossae (asterisks). The lesion shows multiple calcifications (arrows), which are easily identified on CT but also well seen on SWI and can be differentiated from hemorrhage (calcifications are black on magnitude and white on phase maps of SWI in the “right-handed” system, as here).

ACPs typically present as multicystic, multilobulated lesions with calcification and enhancement of the lesion walls and solid components. These tumors follow the classic “90% rule,” in which 90% of lesions show calcifications, cysts, and contrast enhancement [6]. The cysts often contain a dark, “motor oil-like” fluid rich in cholesterol crystals, which appears hyperdense on CT and bright on T1-weighted MR images because of high protein content, cholesterol crystals, or methemoglobin and is not completely suppressed on FLAIR imaging [40]. A level due to different densities can frequently be observed inside the cyst fluid. After gadolinium administration, the enhancement of the cyst walls and solid components is typical (Figure 4). ACPs can be exceptionally large and extend into multiple cranial fossae, with supra- and/or infradiaphragmatic extensions and finger-like projections into adjacent structures [41]. They can also displace and compress nearby intracranial arteries, causing cerebral ischemia (Figure 5). CT remains the gold standard for identifying calcifications, which are thought to result from osteoblastic differentiation of tumor cells [42], although SWI may also provide information about calcifications (Figure 6). The main differential diagnoses include cystic or hemorrhagic pituitary adenomas (rare in children); suprasellar epidermoid, dermoid, and arachnoid cysts (which usually lack peripheral contrast enhancement); and Rathke cleft cysts (typically small, purely cystic, noncalcified lesions without nodular enhancement) [43].

**Figure 7:**
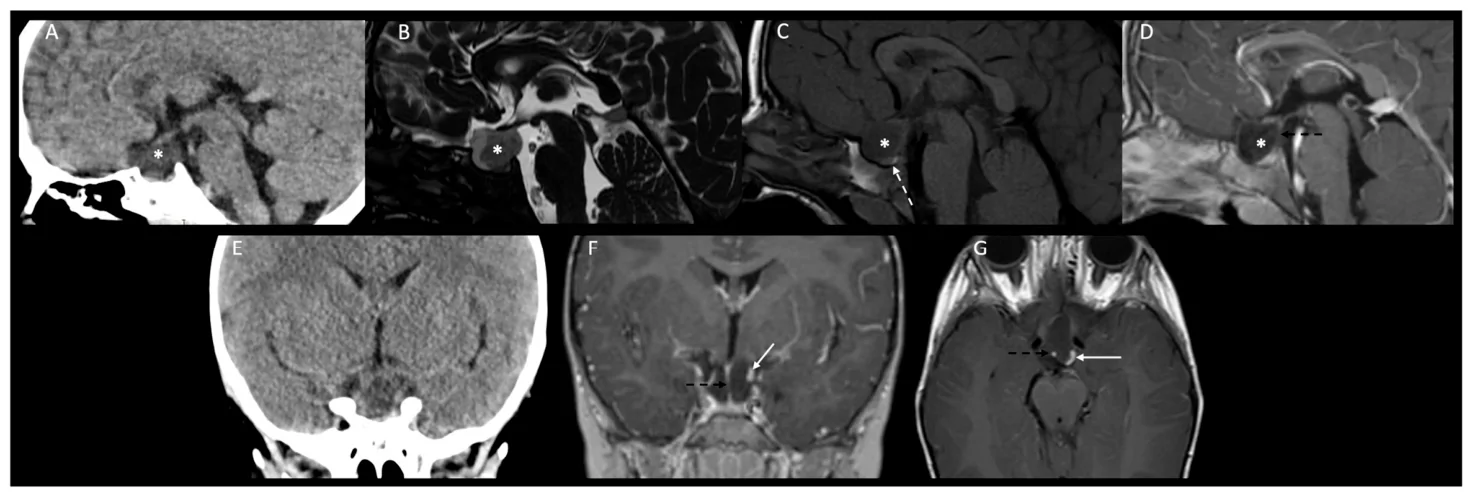
PCP. CT and MR studies of a 7-year-old boy with headache and vomiting. (**A**) Sagittal CT, (**B**) sagittal bSSFP, (**C**) sagittal T1, (**D**) sagittal contrast-enhanced 3D T1, (**E**) coronal CT, (**F**) coronal contrast-enhanced 3D T1, (**G**) axial contrast-enhanced T1. Sellar/suprasellar expansile lesion (asterisks), characterized by inhomogeneous cystic content and strong enhancement of a solid parietal nodule (arrows). Note the absence of calcifications on CT images (**A**,**E**). The pituitary gland is compressed downward (dashed white arrow), and the pituitary stalk appears elongated and stretched posteriorly (dashed black arrows).

PCP are predominantly solid and rarely calcify [44]. On MRI, they typically appear as suprasellar or intraventricular solid, noncalcified masses with smooth margins. Cysts are less common than ACPs are, and when present, they appear as smaller, less complex structures with clear fluid content that is not associated with spontaneous T1-weighted hyperintensity. After gadolinium administration, PCP usually shows relatively homogeneous enhancement (Figure 7). The main differential diagnoses include germ cell tumors, which are characterized by pronounced diffusion restriction, vertical infundibular extension, and pituitary metastases [43].

Beyond histological subtype, topographic MRI-based classification is essential for surgical planning and prognostication. Several studies have demonstrated that MRI-based topographic assessment strongly correlates with surgical approach selection and long-term outcomes [45,46,47,48]. The relevant imaging parameters include third ventricle occupation, pituitary stalk distortion, mammillary body displacement, optic chiasm elevation, and tumor morphology.

Specifically, the standardized staging system proposed by Müller et al. enables the systematic evaluation of tumor relationships along the sellar-suprasellar-third ventricular axis [49]. This grading, based on hypothalamic involvement (HI), is a key determinant of postoperative morbidity, including the risk of hypothalamic syndrome: HI Grade 0: No hypothalamic contact or compression. HI Grade I: Tumor contact or compression of the anterior hypothalamus, sparing the mammillary bodies. HI Grade II: Dislocation, compression, or destruction of the entire hypothalamus, including the anterior structures and mammillary bodies [3].

**Figure 8:**
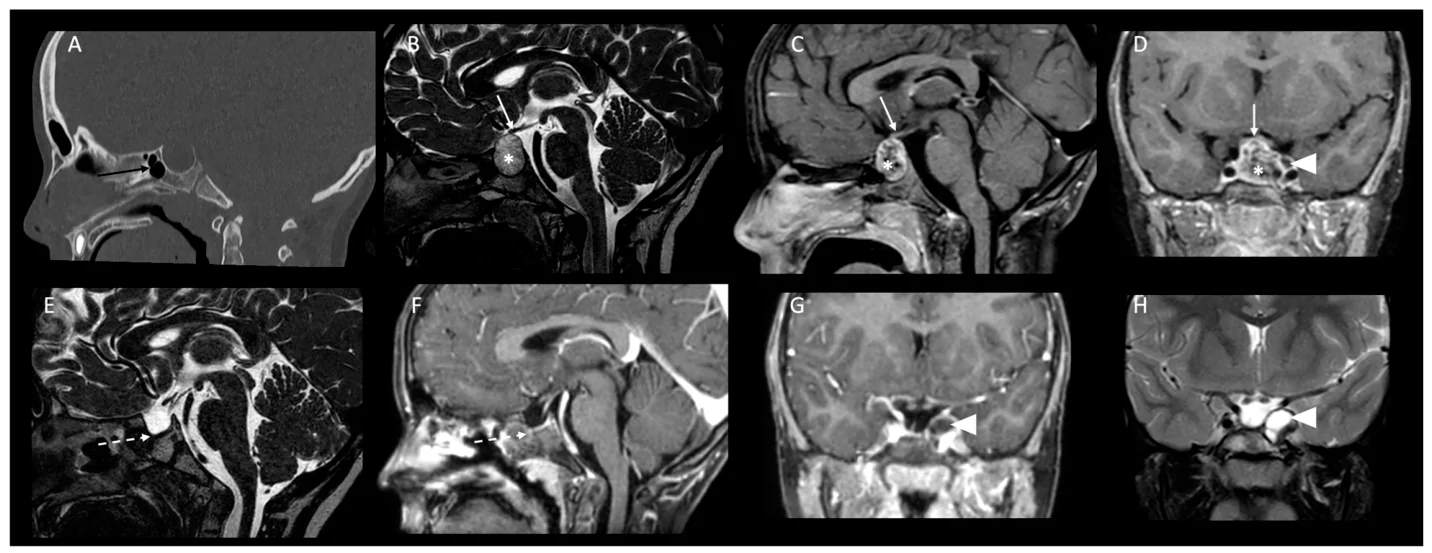
ACP. CT and MRI studies of a 7-year-old boy with headache and worsening vision. (**A**) Sagittal bone CT, (**B**,**E**) sagittal bSSFP, (**C**,**F**) sagittal and (**D**,**G**) coronal contrast-enhanced T1, and (**H**) coronal T2. Sella turcica enlargement due to a sellar-suprasellar multiloculated cystic lesion (asterisks), involving the left cavernous sinus (**D**, arrowhead) and compressing the optic chiasm (white arrows) upward. Note the pneumatization of the sphenoid sinus, best seen on CT (black arrow). Postoperative MRI (**E**–**H**) revealed a transsphenoidal approach (dashed arrow), complete ablation of the sellar–suprasellar lesion, and residual involvement of the left cavernous sinus by a cystic lesion with peripheral enhancement (**G**,**H**, arrowheads).

**Figure 9:**
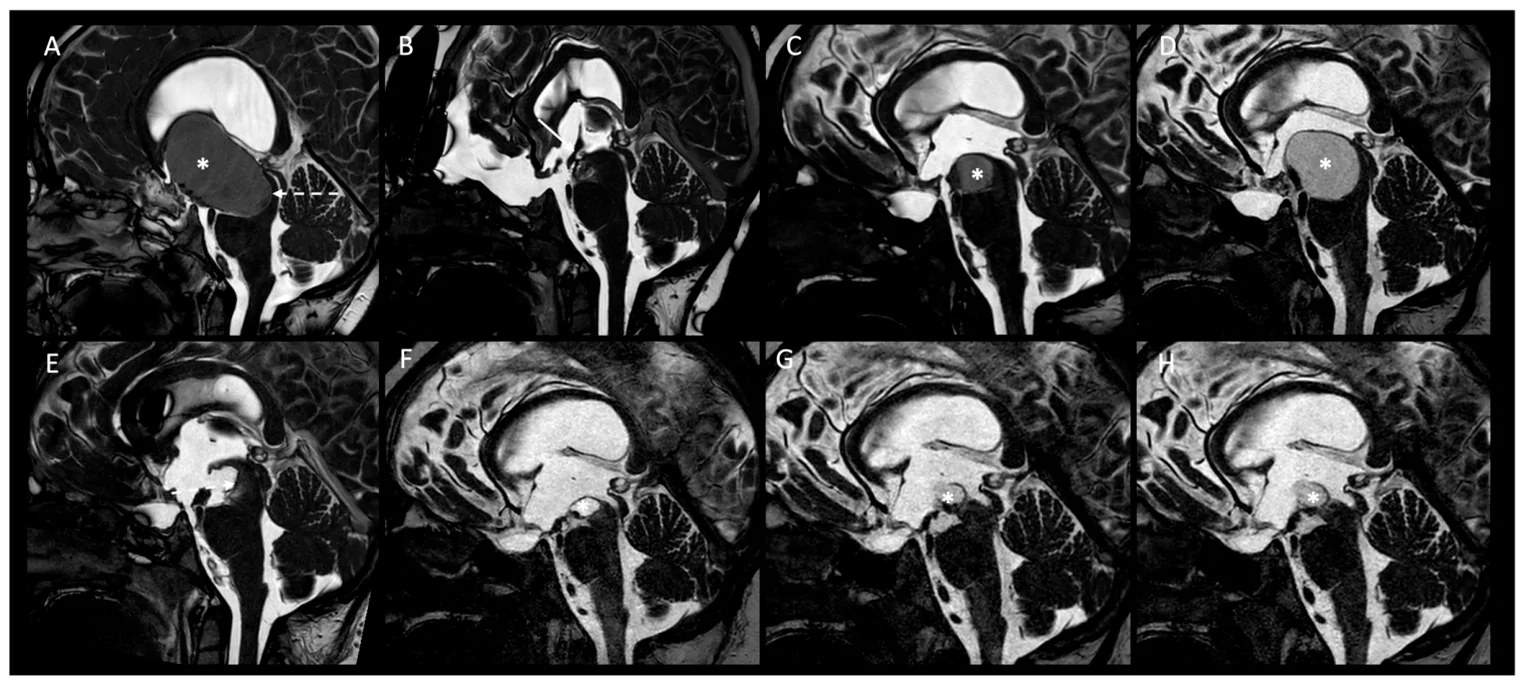
ACP. MRI surveillance using a bSSFP sequence in a 9-year-old boy. MRI at diagnosis (**A**) showing a multiloculated sellar/suprasellar cystic lesion (asterisk) involving both the third ventricle and the interpeduncular cistern with obstructive hydrocephalus and mesencephalic compression (dashed arrow). The first postoperative study (**B**) demonstrates gross total resection of the cystic lesion, with a residual posterior wall adjacent to the mesencephalic floor of the third ventricle (arrow). At the 2-month follow-up (**C**), recurrence of the tumoral cyst involving the third ventricle and the interpeduncular cistern was observed (asterisk). The lesion shows progressive enlargement at 6 months (**D**, asterisk), requiring repeat surgical resection (**E**). At 3 months after the second surgery, a new cystic recurrence was identified at the mesencephalic floor of the third ventricle (**F**, asterisk), which remained stable at the 1-year (**G**, asterisk) and 2-year (**H**, asterisk) follow-ups.

Contrast-enhanced MR remains the reference standard for postoperative assessment and detection of recurrence (Figure 8). However, recent evidence supports the use of noncontrast MRI protocols incorporating three-dimensional high-resolution T2-weighted sequences and bSSFP sequences as reliable alternatives for routine follow-up in selected patients. Given their superior ability to distinguish between soft tissue and CSF within the basal cistern and ventricular system, along with its high signal-to-noise ratio, they are able to effectively differentiate residual craniopharyngiomas from the surrounding CSF, particularly in the suprasellar region where craniopharyngiomas are commonly found. These sequences have shown high concordance with contrast-enhanced T1-weighted imaging in assessing residual or recurrent disease, particularly in long-term surveillance [50] (Figure 9). This is particularly important in pediatric patients, in whom minimizing cumulative gadolinium exposure is desirable.

Pretreatment endocrine evaluation is crucial, including a complete assessment of anterior and posterior pituitary function, whenever the patient’s clinical condition allows and immediate neurosurgical intervention is not required [51]. Detailed neuro-ophthalmologic assessment, including visual field testing, is essential for determining the degree of optic pathway compression and establishing a preoperative baseline.

## 5. Prognosis, Follow-Up and Long-Term Consequences

Pediatric craniopharyngiomas are frequently associated with pituitary hormone deficiencies, which typically exacerbate following therapeutic intervention [52].

Among endocrine deficiencies, the most prevalent is growth hormone deficiency (GHD), affecting 26–75% of patients at diagnosis and increasing to 40–100% posttreatment [53]. In very obese patients, short stature can be absent, and clinical suspicion must be raised. Treatment with recombinant growth hormone should be initiated as early as possible to minimize the auxological and metabolic consequences of GHD. Recent studies have confirmed the safety of GH treatment in these patients, and in very severe cases, treatment can be started after three months of tumor stability [54].

Hypogonadotropic hypogonadism is commonly already present at CP diagnosis, occurring in more than 20% of cases, but its prevalence increases to 80% following treatment [55,56]. Appropriate puberty induction and sex hormone replacement therapy are extremely important to allow optimal bone health during adulthood, particularly in women, as well as to prevent unfavorable metabolic outcomes.

ACTH deficiency is a frequent endocrine complication of craniopharyngioma treatment, with a prevalence of 29–96.8% [56]. Replacement treatment should be initiated before any other endocrine treatment to avoid adrenal crisis [57]. Patients may benefit from modified-release hydrocortisone, which can also enhance adherence to treatment. In particular, recent data from the CHAMPAIN trial demonstrate that [58] nocort—a formulation that restores the physiological early-morning cortisol peak—significantly improves quality of life, reduces morning fatigue, and enhances immune profiles compared to Plenadren, which only provides daytime cortisol coverage for patients with adrenal insufficiency. Although these findings cannot be directly extrapolated to patients with craniopharyngioma and secondary adrenal insufficiency, they highlight the potential benefits of more physiological glucocorticoid replacement strategies, which warrant further investigation in this population.

CDI is also common in patients with AVP-D, presenting after treatment in 60–90% of children and 50–70% of adults [59]. Careful monitoring of fluid balance and electrolytes following craniopharyngioma surgery is essential to avoid life-threatening electrolyte fluctuations and to establish the most appropriate desmopressin regimen.

HS, due to hypothalamic damage caused by the tumor itself and/or by surgery, comprises a spectrum of symptoms, including hypothalamic obesity with disordered eating and hyperphagia, neurocognitive and behavioral changes (memory deficits, emotional lability, depression), sleep disturbances, fatigue, and autonomic dysfunction (imbalances in body temperature, thirst, heart rate, and blood pressure regulation). The prevalence of HS is approximately 35% at diagnosis but increases to 65–80% after surgery, particularly after gross total resection [60,61]. Recently, a novel diagnostic scoring system has been developed to improve the early recognition and assessment of HS in pediatric cohorts. This system defines diagnostic criteria across three domains—clinical, radiological, and pre-test probability—and categorizes symptoms into minor and major criteria to evaluate the presence of the syndrome [62].

Glucagon-Like Peptide-1 Receptor Agonists (GLP-1RA)and setmelanotide, a melanocortin receptor agonist, have recently demonstrated potential in addressing hypothalamic obesity [63,64,65]. In addition, dexamphetamine has demonstrated clinical utility in managing hypothalamic obesity, primarily by curbing hyperphagia, which leads to sustained weight loss or stabilization in patients with craniopharyngioma [3].

Disruptions in sleep and cognitive decline are frequently neglected. In clinical practice, they should be consistently evaluated in patients with hypothalamic involvement and promptly treated [66,67].

Cardiovascular and bone health issues must be actively managed to improve long-term results [68].

This high morbidity associated with CP often results in a variety of physical, cognitive, and psychological deficits that affect quality of life (QoL) in the long term. Both early endocrine dysfunction replacement and hypothalamic obesity treatment, together with routine neuropsychological and psychosocial assessments, will contribute to ensuring long-term well-being.

## 6. Treatment Modalities

### Surgical Management

**Figure 10:**
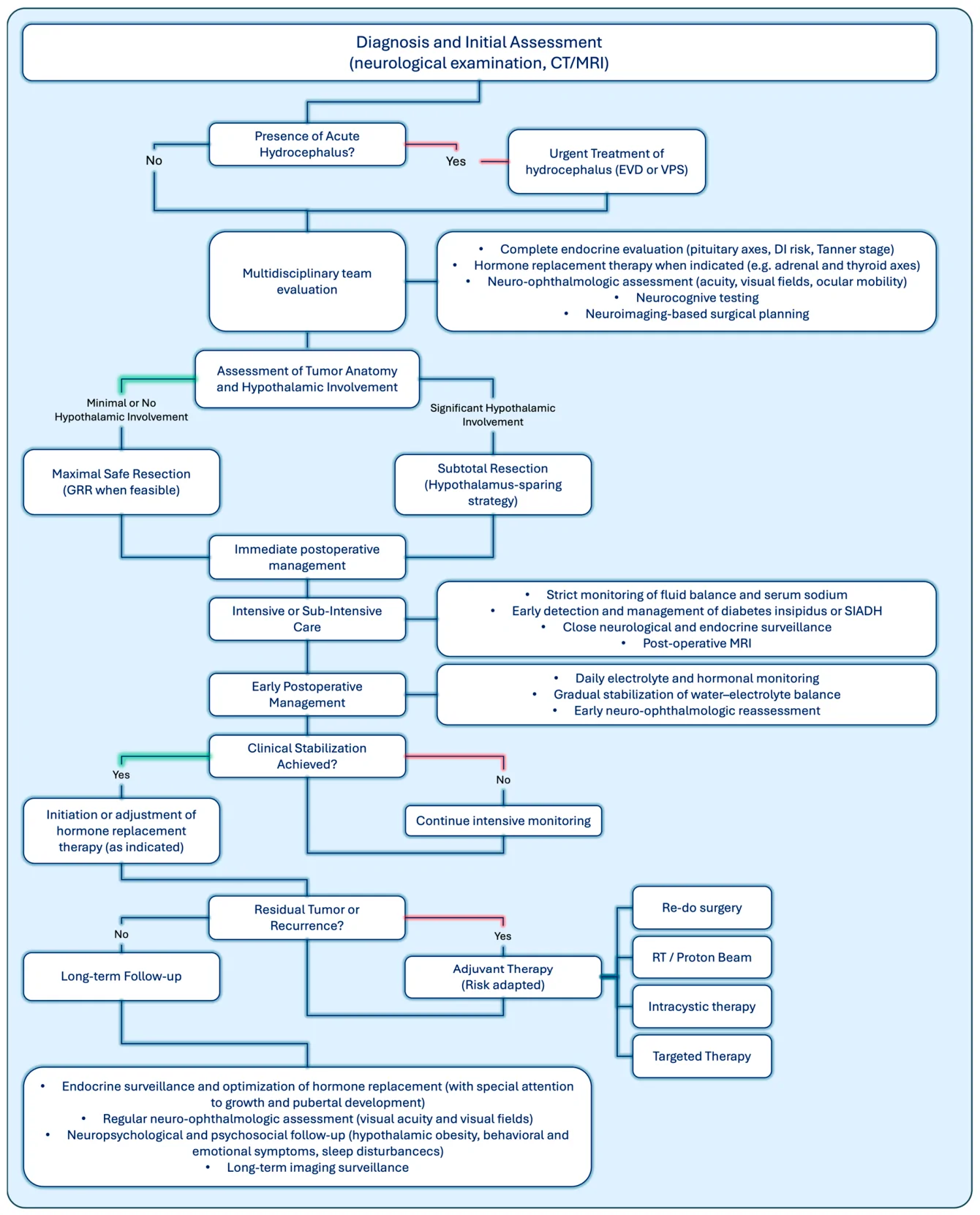
Risk-adapted, multidisciplinary treatment algorithm for pediatric craniopharyngiomas. SIADH: Syndrome of Inappropriate Antidiuretic Hormone Secretion.

Surgery remains the cornerstone of initial management for pediatric craniopharyngiomas, with the goal of obtaining histological confirmation, decompressing critical neurovascular structures, and achieving durable disease control. In recent decades, surgical philosophy has progressively evolved from aggressive radical resection to risk-adapted strategies that prioritize the preservation of hypothalamic, endocrine, and neurocognitive functions (Figure 10).

Gross total resection (GTR) was historically regarded as the gold standard for the treatment of craniopharyngiomas, with early surgical strategies favoring aggressive resection aimed at complete tumor removal to achieve long-term cure. Although GTR was initially supported by excellent tumor control rates and low recurrence rate [69,70,71,72], accumulating long-term evidence has shown that it does not confer a significant progression-free survival advantage over less radical approaches, while being associated with a substantially higher risk of hypothalamic injury and long-term functional morbidity. In particular, Sterkenburg et al. [73] reported comparable progression-free survival after incomplete resection, highlighting that preservation of hypothalamic function should be prioritized whenever feasible.

However, aggressive attempts at complete tumor removal—particularly in lesions with posterior extension or hypothalamic adherence—carry a substantial risk of severe and often irreversible morbidity, including hypothalamic obesity, panhypopituitarism, neurocognitive impairment, and behavioral disturbances [17,74,75]. Consequently, contemporary surgical strategies advocate reserving GTR for tumors without or with minimal hypothalamic involvement, as defined by risk stratification systems such as the Puget classification (grade 0–1) [17].

Subtotal resection (STR) has therefore emerged as the preferred approach for tumors with significant hypothalamic involvement (Puget grade 2) [17,49,75]. The primary goal of STR is maximal safe debulking to relieve compression of the optic apparatus and third ventricle while minimizing hypothalamic injury. Although STR alone is associated with high rates of progression, its combination with adjuvant radiotherapy provides long-term tumor control comparable to that achieved with GTR, with a significantly reduced burden of treatment-related morbidity [74,76,77]. This strategy reflects the current paradigm shift toward functional preservation rather than anatomical radicality.

In parallel to this conceptual evolution, surgical techniques have undergone significant refinement. The endoscopic endonasal approach (EEA) has become an increasingly valuable option in selected pediatric patients and represents a minimally invasive alternative to traditional transcranial routes.

EEA is particularly well suited for midline craniopharyngiomas with a pure intrasellar extension or with a suprasellar component contained underneath a competent diaphragma sellae [9,78,79,80]. In such configurations, the endonasal corridor provides a direct midline trajectory, prevents brain retraction, and allows early visualization and decompression of the optic nerves and chiasm. Approaching the lesion from below may facilitate the identification of tumor–hypothalamic interfaces and potentially reduce manipulation of posterior hypothalamic structures.

In contrast, EEA is less appropriate for tumors with marked superior extension into the third ventricle, significant posterior hypothalamic involvement, or lateral extension beyond the carotid arteries and cavernous sinus, where the anatomical limits of the endonasal corridor may compromise surgical safety or completeness [81,82]. Therefore, large, multicompartmental tumors with extensive lateral or posterior growth are often better managed through transcranial routes [72].

Systematic analyses comparing transcranial and endoscopic approaches in children suggest a clear evolution in surgical practice rather than a universal superiority of one technique over the other. A recent systematic review [83] demonstrated that the EEA can achieve extent-of-resection and progression-free survival rates comparable to those of transcranial surgery in carefully selected pediatric patients, emphasizing the importance of anatomical selection criteria and surgical expertise [84,85]. Importantly, rates of endocrine dysfunction remain high across approaches, reinforcing the concept that hypothalamic involvement—rather than surgical route—is the principal determinant of long-term morbidity [86].

Despite its advantages, the EEA presents unique challenges in the pediatric population. These include limited sphenoid sinus pneumatization in younger children, narrow nasal corridors, an increased risk of postoperative cerebrospinal fluid (CSF) leakage, and concerns regarding skull base reconstruction in a growing craniofacial skeleton [87,88,89]. Furthermore, the learning curve for the pediatric EEA is steep, and surgical outcomes are highly dependent on multidisciplinary expertise and institutional volume [69,70]. Nevertheless, growing evidence has indicated that in experienced hands and with the adoption of modern skull base reconstruction techniques [90], these risks can be significantly mitigated, and the anatomical limitations of the endonasal corridor become less restrictive [91,92]. As a result, when performed in specialized high-volume centers, pediatric EEA has demonstrated progressively improved safety profiles and expanding indications [83].

Despite the growing adoption of endoscopic techniques, microsurgical transcranial approaches continue to play a pivotal role in the surgical management of pediatric craniopharyngiomas [72,93]. Traditional routes, including pterional and supraorbital (eyebrow) approaches, offer wide and flexible exposure of the suprasellar region, optic nerves, hypothalamus, and third ventricle, allowing meticulous microsurgical dissection under direct, multiangled visualization [94]. These approaches are particularly advantageous for tumors with substantial superior extension into the third ventricle, posterior hypothalamic involvement, or lateral growth beyond the carotid arteries and cavernous sinus, anatomical configurations in which the endoscopic endonasal corridor may be restricted or unsafe [81,82,93]. Moreover, transcranial microsurgery facilitates early proximal vascular control and safer handling of complex neurovascular relationships, especially in large, multicompartmental, or laterally extended lesions [82]. Nevertheless, transcranial approaches are inherently associated with potential limitations, including the need for brain retraction and the risk of manipulation-related injury to critical neural structures, underscoring the importance of careful patient selection and surgical expertise.

Minimally invasive variants, such as the supraorbital approach, have been proposed in selected cases to reduce surgical morbidity while preserving the advantages of microsurgical visualization [95,96,97], although their applicability remains limited to carefully selected anatomical configurations in the presence of specific surgical expertise.

## 7. Radiation Therapy

The choice between adjuvant radiotherapy and observation is primarily determined by the presence of residual disease and the patient’s age. In very young patients, greater susceptibility to the adverse effects of radiation warrants observation. However, lesions undergoing incomplete resection have a high probability of local recurrence in the absence of adjuvant therapy (71–90%), suggesting that postoperative radiotherapy is recommended in cases of subtotal resection (STR) [98,99].

Numerous radiotherapy modalities have demonstrated excellent results in terms of local control, although prospective studies comparing the efficacy of different advanced photon-based and proton-based technologies in reducing adverse effects are lacking. The available literature is mainly based on retrospective series employing photon radiotherapy, either conventional or hypofractionated stereotactically [100,101,102,103,104,105].

Prospective and retrospective studies have documented the efficacy of three-dimensional conformal radiotherapy (3D-CRT) with reduced margins [101], intensity-modulated radiotherapy (IMRT) [106], and volumetric modulated arc therapy (VMAT), with the latter paying particular attention to minimizing hippocampal exposure. Gamma Knife radiosurgery, reserved for small lesions (<3 cm) with adequate distance from critical structures, has shown satisfactory control rates with single-fraction doses of 12–14 Gy [107,108,109,110,111,112], although the limited sample sizes and heterogeneity of selection criteria make comparisons with conventional fractionated regimens complex.

Proton therapy is assumed to play an increasing role because of its dosimetric advantages in sparing vascular structures and the hippocampus, particularly with advanced modalities such as intensity-modulated proton therapy (IMPT) [113]. Comparative studies have documented equivalent clinical outcomes between proton therapy and photon radiotherapy [114,115].

## 8. Radiotherapy Details and Target Volume Definition

In conventional fractionation schedules, the doses used range between 50.4 and 54 Gy in 1.8 Gy fractions, with the upper limit reflecting the tolerance of critical structures such as the optic chiasm [100,101,115,116]. Modern photon techniques (IMRT and VMAT) have improved the sparing of healthy tissues, with progression-free survival rates comparable to those of 3D-CRT [106].

**Figure 11:**
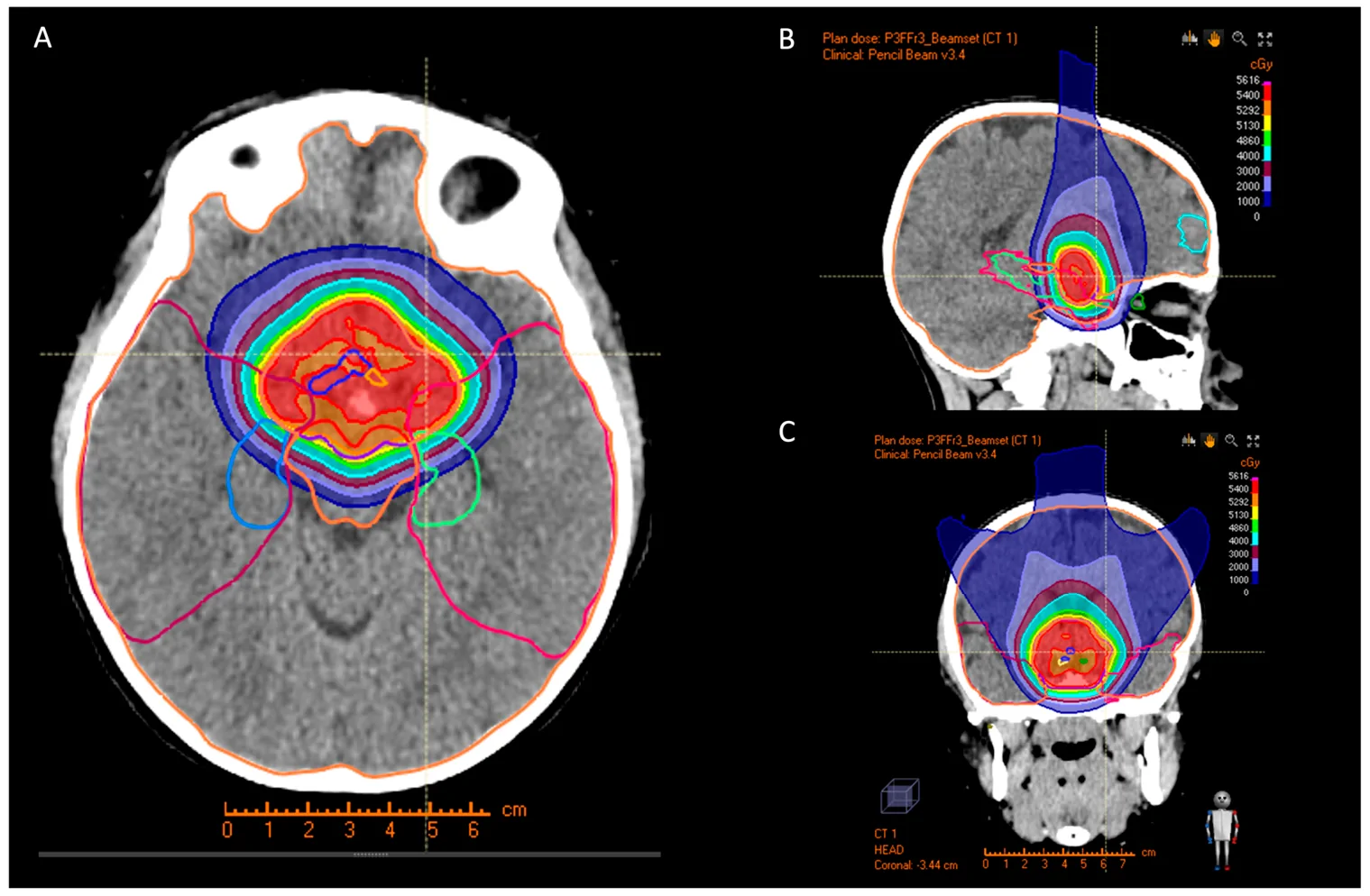
Proton beam therapy treatment plan for pediatric craniopharyngioma. Distribution of representative proton therapy doses displayed on planning CT images in the axial (**A**), sagittal (**B**), and coronal (**C**) planes. Color-coded isodose lines illustrate conformal target coverage, with the highest dose concentrated within the planning target volume (PTV) and a steep dose falling off beyond the target.

With respect to the target volume definition, the gross tumor volume (GTV) is generally well delineated when MR-CT fusion is used (Figure 11). It is recommended to include the entire cystic volume in the GTV, given the possible presence of neoplastic cells in the cyst wall. The expansion of the clinical target volume (CTV) is a subject of debate: historically, margins of 1–2 cm were used, but recent studies suggest that a reduced expansion of 5 mm may be safe, given the noninfiltrative nature of craniopharyngioma. The CTV definition must nonetheless be personalized, including areas of tumor adherence identified intraoperatively. According to institutional practices, the planning target volume (PTV) in photon radiotherapy involves expansions that typically range between 3 and 5 mm. Cystic components require particular attention because of their dynamic nature. Studies have documented cystic expansion during radiotherapy in a significant proportion of patients (up to 40%), with the need for plan revision in 20% of cases [115,117]. Therefore, weekly or biweekly imaging monitoring during treatment is recommended in patients with a cystic component, especially when reduced margins are used or when proton therapy is employed.

## 9. Emerging Targeted Therapies

The progressive elucidation of the molecular and biological underpinnings of craniopharyngiomas has prompted the development of targeted and biologically driven therapeutic strategies, particularly in the context of recurrent or progressive diseases. However, the clinical applicability of these approaches differs substantially between the PCP and ACP subtypes, reflecting their distinct oncogenic drivers and tumor microenvironments (Figure 2) [118,119].

PCP is characterized by a high prevalence of activating BRAF V600E mutations, which result in constitutive activation of the MAPK/ERK signaling pathway. This molecular alteration provides a robust and clinically actionable target. Consequently, systemic inhibition of the MAPK pathway using clinically available BRAF inhibitors, such as dabrafenib or vemurafenib, in combination with MEK inhibitors, such as trametinib or cobimetinib, has emerged as the most mature example of targeted therapy in craniopharyngioma [118,119]. Clinical experiences, primarily derived from adult cohorts but increasingly extended to adolescent patients, have consistently demonstrated rapid and often profound tumor regression. These responses have stimulated interest in the use of BRAF–MEK inhibition not only for recurrent or unresectable disease but also in neoadjuvant settings to reduce tumor volume and potentially facilitate less morbid surgical or radiotherapeutic interventions [118].

The clinical relevance of this approach is currently being assessed in prospective trials. The Alliance A071601 study (NCT03224767), a phase II trial, is evaluating combined vemurafenib and cobimetinib in patients with BRAF V600E–mutant craniopharyngioma, while the SWECRANIO trial (NCT05525273), also phase II, is investigating dabrafenib plus trametinib specifically in papillary craniopharyngioma [118,119]. Although early results support the biological efficacy of MAPK pathway inhibition, critical questions remain regarding the optimal treatment duration, long-term disease control after treatment discontinuation, and potential late toxicity, particularly in the pediatric population, where prolonged pathway suppression raises concerns related to growth and development [119].

In contrast, ACP is driven by activating mutations in CTNNB1, leading to aberrant stabilization and nuclear accumulation of β-catenin and dysregulated Wnt/β-catenin signaling. Direct pharmacological targeting of this pathway remains unfeasible with current agents because of toxicity and lack of specificity [118,119,120]. As a result, therapeutic research on ACPs has shifted toward indirect strategies informed by the complex biology of tumors, including intratumoral heterogeneity, paracrine signaling, and a pronounced inflammatory microenvironment. Transcriptomic and proteomic analyses have consistently highlighted the upregulation of inflammatory mediators, particularly interleukin-6 (IL-6), as well as the activation of downstream signaling pathways such as MAPK/ERK in subsets of tumors [119,120].

These observations have provided the rationale for several ongoing clinical trials. MEK inhibition is being explored in the CONNECT2108 trial (NCT05286788), a phase II study evaluating binimetinib in children and young adults with progressive or recurrent ACP [118,119]. In parallel, targeting tumor-associated inflammation has emerged as a complementary strategy. Systemic blockade of the IL-6 receptor with tocilizumab is currently under investigation in a phase II trial (CONNECT1905; NCT05233397), following earlier feasibility data obtained in an early phase 1 study (NCT03970226) [119,120,121]. Additionally, the PNOC029/ITCC-118 trial (NCT05465174), a phase I–II study, is assessing a combined approach using tovorafenib together with immune checkpoint blockade, reflecting a growing effort to integrate molecularly targeted and immunomodulatory strategies in ACP [118,119]. At present, these approaches remain investigational.

## 10. Intracystic Therapies

Intracystic therapies remain a cornerstone adjunctive strategy in the management of pediatric craniopharyngiomas, particularly in ACPs, where cystic components are frequent and often dominate the clinical course [120,121]. Effective cyst control may substantially influence symptoms, the timing of surgery, and the exposure of critical hypothalamic and optic structures to aggressive treatments. Intracystic therapies require the surgical implantation of an intracystic catheter connected to an Ommaya reservoir, allowing repeated cyst aspiration to relieve mass effect and intracystic administration of therapeutic agents without the need for repeated surgical procedures. In addition to facilitating drug delivery, intermittent reservoir puncture can provide effective decompression of enlarging cysts and symptomatic relief while preserving surrounding neurovascular structures.

Historically, intracystic agents such as bleomycin and radioisotopes, including phosphorus-32, have been used to induce cyst sclerosis. However, concerns regarding neurotoxicity related to intracystic leakage, variable efficacy, and logistical constraints have led to a gradual decline in their use, and these modalities are now largely confined to selected cases or historical series [120].

In contemporary pediatric practice, intracystic interferon-α (IFN-α) has emerged as the most widely adopted and best-characterized type of intracystic therapy. IFN-α exerts immunomodulatory, antiproliferative, and proapoptotic effects that are particularly suited to the inflammatory epithelial lining of ACP cysts. Long-term institutional experiences and cooperative group studies, including the PBTC-039 phase II trial (NCT01964300) evaluating pegylated interferon-α, have demonstrated that this approach is generally well tolerated and can achieve meaningful cyst reduction or stabilization in a substantial proportion of patients [120]. Importantly, intracystic IFN-α may allow deferral of surgery or radiotherapy, particularly in younger children, although disease control is frequently temporary and cyst recurrence remains common [120].

More recently, attention has focused on targeting inflammatory mediators, particularly IL-6, which is abundantly expressed in ACP cyst fluid, within the cystic microenvironment. On this basis, tocilizumab has been explored via systemic and intracystic or intratumoral administration. Early prospective pediatric series have reported acceptable safety but variable and often limited cyst control at the tested dosing schedules, emphasizing the need for further optimization and biomarker-driven patient selection [119,120,121]. At present, IL-6–directed intracystic therapy should be regarded as experimental and pursued within clinical trials or highly selected refractory cases managed at specialized centers.

In summary, intracystic therapies play important roles in controlling the cystic disease burden and alleviating mass effects, thereby contributing to functional preservation. Their use exemplifies the broader shift toward individualized, morbidity-sparing treatment strategies in pediatric craniopharyngioma [120,121].

## 11. Outcomes

Despite the high rates of local control, the 10-year overall survival of patients with craniopharyngioma ranges between 80% and 91%, which is lower than that of the general population, with an increased risk of mortality from cardiovascular and cerebrovascular causes [59,115,116,117,122,123]. No significant differences in long-term survival were observed between patients who underwent GTR and those who underwent STR followed by RT. GTR rates vary widely (30–75%), with recurrence occurring in 8–30% of patients after GTR [71,124,125,126,127,128]. After STR alone without radiotherapy, the recurrence rate is unacceptably high (40–75%) [71,123,129], whereas STR followed by postoperative radiotherapy ensures control rates comparable to those of GTR (0–12% recurrence) [115,116,117]. Progression of cystic components is more frequent and more difficult to control than that of solid components (3-year recurrence rates: 24% vs. 5%) [115], although the increase in cyst size during the postradiotherapy period is often transient [115]. In the event of recurrence, surgical management is complex because of increased adherence to surrounding tissues, with reduced resection rates (13–53%) [71,123,129,130]. Compared with surgery alone, the combination of surgery and radiotherapy ensures durable control (10-year progression-free survival: 82–83%) [131]. Therefore, omitting radiotherapy in definitive treatment with the intent of reserving it for salvage is not justified, as recurrent cystic regrowth is associated with worse visual outcomes, hypothalamic obesity, and worsening of endocrine function [115].

## 12. Toxicity

Treatment of craniopharyngioma is associated with a significant burden of morbidity and is often difficult to distinguish from sequelae of the disease itself. Endocrine dysfunction is the most common toxicity, with transient diabetes insipidus occurring in 80–100% of patients and permanent AVP-D occurring in 40–93% of patients [70,124,132,133]. Panhypopituitarism is observed in up to 75–100% of patients after surgery [14,71,131] and in 30–46% of patients after radiotherapy [115,134]. Visual outcomes may improve (41–58%) or worsen (2–66%) following resection [71,72,124,133,135]. Hypothalamic dysfunction, often underestimated, manifests as alterations in circadian rhythm, behavior, obesity, and disturbances in thermoregulation and thirst, worsening in 65–80% of patients [72,75,134]. Hypothalamic obesity, which requires specialist management, has a prevalence of 25–55% after multimodal treatment [72,115,136]. Neurocognitive decline is more pronounced after aggressive surgery alone (mean IQ reduction of 10 points) than after surgery combined with radiotherapy (1.25 points) [132]. Cerebrovascular changes are associated with a tenfold greater risk of stroke in pediatric brain tumor patients than in the general population [137], and the prevalence of vasculopathies is as high as 32% in long-term craniopharyngioma survivors [138,139,140]. Modern radiotherapy techniques, owing to better dose conformity and sparing of healthy tissues, may reduce the incidence of such late toxicities compared with historical cohorts.

## 13. Future Directions

Future developments in the management of pediatric craniopharyngiomas will focus increasingly on personalized, risk-adapted strategies aimed at minimizing long-term morbidity while preserving durable tumor control. The integration of molecular profiling into clinical practice, particularly the identification of actionable alterations such as BRAF V600E in papillary craniopharyngiomas, is expected to further influence therapeutic sequencing and may allow for de-escalation in selected cases.

Advances in neuroimaging and radiomics may enhance the preoperative assessment of hypothalamic involvement and support more accurate prediction of treatment-related outcomes, facilitating anatomy-driven surgical decision-making. In parallel, continued refinement of minimally invasive surgical techniques and precision radiotherapy approaches aims to reduce approach- and radiation-related toxicity.

Finally, the establishment of prospective multicenter registries and long-term survival studies remains essential to better define late effects, quality-of-life outcomes, and neurocognitive trajectories, ultimately guiding evidence-based, multidisciplinary care for this rare type of pediatric tumor.

## 14. Conclusions

Treating craniopharyngiomas in children requires a nuanced balance between oncologic efficacy and functional preservation. While surgery remains the cornerstone of management, adjuvant radiotherapy and intracystic therapies expand the therapeutic armamentarium and support a multidisciplinary approach. Advances in surgical techniques and treatment planning have shifted contemporary strategies toward risk-adapted, anatomy-driven decision-making aimed at minimizing long-term morbidity. Integrating molecular insights, advanced imaging, and individualized treatment strategies will be essential to further reduce the lifelong burden associated with this tumor.

## Data Availability

No new data were created or analyzed in this study. Data sharing is not applicable to this article.
